# Genomic analysis reveals convergent signatures of selection for milk traits in sheep and goats

**DOI:** 10.1186/s40104-025-01334-2

**Published:** 2026-02-06

**Authors:** Zhanerke Akhatayeva, Yilong Shi, Kairat Dossybayev, Nurlan Malmakov, Hairong Cheng, Narantuya Baatar, Ji Yang, Menghua Li, Kejian Lin, Songsong Xu

**Affiliations:** 1 Institute of Grassland Research, Chinese Academy of Agricultural Sciences, Hohhot, 010010 China; 2 LPP “Kazakh Research Institute for Livestock and Fodder Production”, Almaty, 050035 Kazakhstan; 3 College of Animal Science and Technology, China Agricultural University, Beijing, 100193 China; 4 Ordos Agricultural and Pastoral Technology Promotion Center, Ordos, Inner Mongolia Autonomous Region 017200 China; 5 Research Institute of Animal Husbandry, Ulaanbaatar, 17029 Mongolia

**Keywords:** Breeding, Convergent evolution, Goat, Milk traits, Sheep

## Abstract

**Background:**

Convergent evolution offers a unique lens through which to explore the molecular underpinnings of significant phenotypic transformations. Similar selective pressures likely drove the evolution of analogous milk traits in sheep and goats. Consequently, the current study aimed to identify common selection signals for milk traits across dairy and non-dairy breeds of sheep and goats worldwide.

**Results:**

In this study, a total of 308 whole-genome sequences from diverse sheep (*n* = 108) and goat (*n* = 200) breeds, including both dairy and non-dairy types, across the world were utilized. The population structure and genetic diversity of dairy and non-dairy sheep and goat breeds were characterized. Species-specific genes associated with milk traits, such as *POU2F1*, *ABCD2*, *TRNAC-GCA* in sheep and *PRPF6*, *VPS13C*, *TPD52L2*, *NFX1* and *B4GALT1* in goats, were identified. Further gene annotation and bioinformatics analyses indicated that different biological pathways are important for milk traits in each species: fatty acid oxidation and AMP metabolic process in sheep, the U2-type spliceosomal complex and propanoate metabolism in goats. Additionally, common signatures of selection such as *CLASP1*, *PDS5B*, *ZNF831*, *CCDC73* were found in sheep and goats. Haplotype and transcriptional analyses further confirmed the role of these genes in milk production and provided evidence for their analogous evolution in sheep and goats. The *CLASP1 *gene was identified as a target of convergent selection, representing a promising candidate for genetic improvement programs in dairy species.

**Conclusions:**

These results provide insights into the genetic basis of convergent dairy traits, offering valuable targets for improving milk production and advancing dairy sheep and goat breeding programs.

**Supplementary Information:**

The online version contains supplementary material available at 10.1186/s40104-025-01334-2.

## Background

Convergent evolution describes the independent acquisition of analogous traits by phylogenetically distinct lineages facing comparable selective pressures, often reflecting parallel responses to shared ecological constraints. Despite lacking recent shared ancestry, this evolutionary convergence results in phenotypic analogues such as morphological, physiological, or behavioral traits that fulfill convergent functional roles across divergent taxa [1, 2]. Investigating convergence signatures in domestic animals provides critical insights into how conserved molecular mechanisms mediate species-specific adaptations to both natural environmental stressors and anthropogenic selection pressures. For example, comparisons of livestock genomes have revealed strong signals of convergent selection in genes involved in melanin metabolism [3, 4]. Likewise, previous studies have identified specific genetic variants such as chr14:19947421 in *ASIP* and chr25:T7068586C in *IRF2BP2* that distinguish domesticated populations from their wild ancestors in cattle [5] and sheep [6], respectively. Deciphering these genetic parallels ultimately enables the development of predictive breeding frameworks that leverage evolutionary principles for targeted trait optimization.

Sheep and goat breeding plays an essential role in the livelihoods of a global population. As world’s population growth increases the demand for agricultural resources, there is a need to expand livestock production [7]. Notably, sheep and goats evolved from their last common ancestors *Ovis orientalis* and *Capra aegagrus* about 10,000–11,000 years ago, offering a compelling case of convergent evolution [8–10]. Over centuries, domestication and selection have significantly reshaped their genomes to meet diverse environmental challenges and human needs. After plenty of years of adaptation to local conditions, there are now a large number of goat and sheep breeds that are adapted to the domestic conditions, some of them to extreme harsh circumstances [11]. Alberto et al. [9] investigated convergent evolution during the domestication of sheep and goats, identifying shared genomic regions associated with neural, immune, and production-related traits between *Capra hircus* and *Ovis aries*. It was found that nearly half of the genes under selection in sheep also showed selection signatures in goats. More recently, Yang et al. [12] reported convergent evolution in key genes such as *BMPR1B* and *BMPR2*, which are associated with reproductive traits in sheep and goats. In light of rising interest in milk production from small ruminants [13], research has increasingly focused on uncovering the genetic potential of dairy sheep and goats. Although studies on milk traits have employed genome-wide association analyses [14, 15], selection signatures [16, 17], and transcriptome profiling [18, 19], few have explicitly addressed genetic convergence related to milk traits in sheep and goats. We therefore hypothesized that sheep and goats may have evolved similar genetic mechanisms underlying milk production through convergent selection. The present study aimed to characterize genetic diversity and population structure across dairy and non-dairy sheep and goats, as well as to identify shared genomic regions associated with milk production traits in these two closely related species.

## Methods

### Data collection

**Fig. 1:**
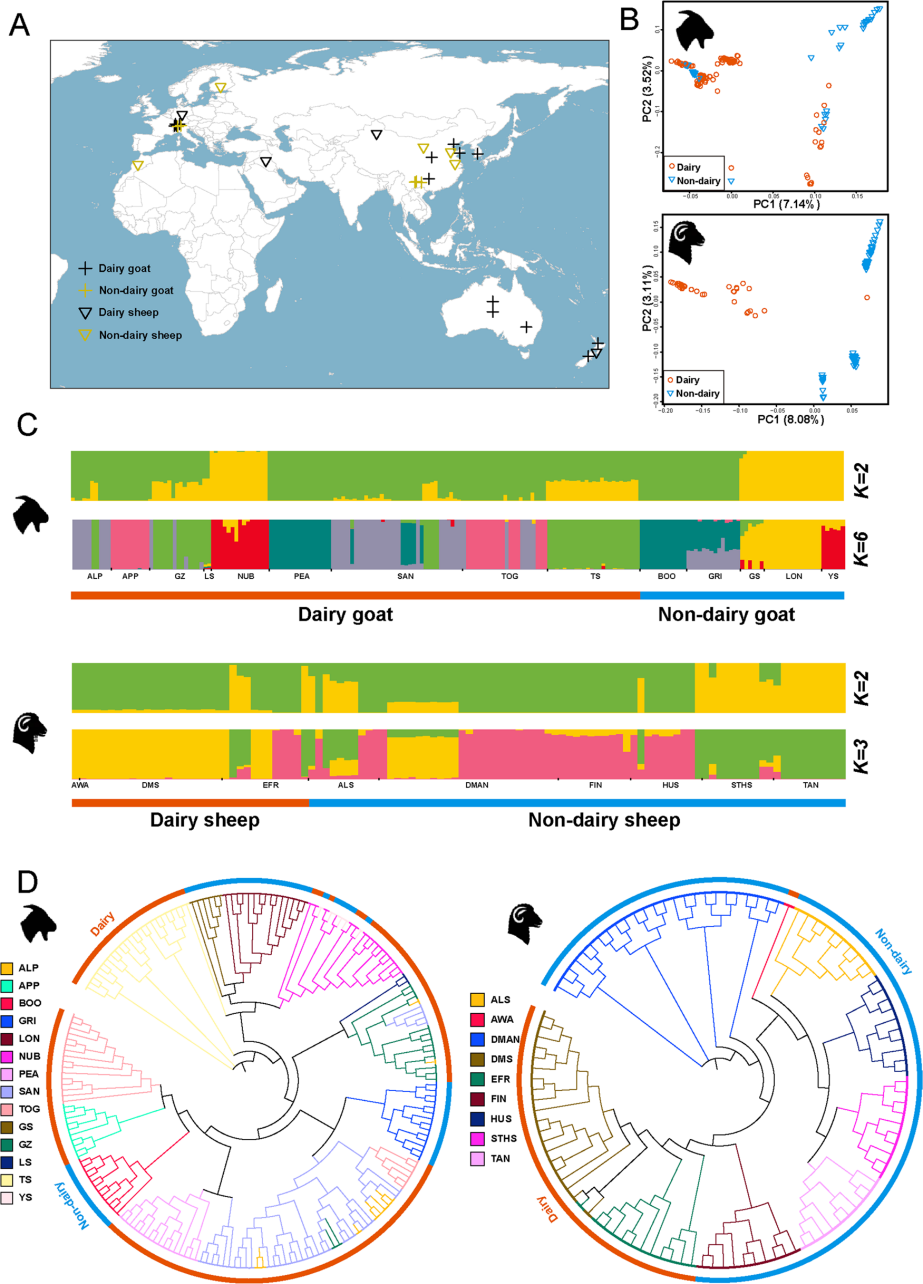
Population structure of goats and sheep. **A** The geographic distribution of dairy and non-dairy goat and sheep breeds. **B** Principal component analysis (PCA) of dairy and non-dairy goats and sheep. **C** Structure analysis for goats and sheep. **D** Phylogenetic tree analysis of goats and sheep. ALP, Alpine goats; APP, Appenzell goats; BOO, Booted goats; GRI, Grisons Striped goats; GS, Guishan goats; GZ, Guanzhong dairy goats; LS, Laoshan dairy goats; LON, Longling goats; NUB, Nubian goats; PEA, Peacock goats; SAN, Saanen dairy goats; TOG, Toggenburg goats; TS, Tangshan dairy goats; YS, Yunshang black goats. ALS, Altay sheep; AWA, Awassi sheep; DMAN, D'man sheep; DMS, Dairy Meade sheep; EFR, East Frisian dairy sheep; FIN, Finnish sheep; HUS, Hu sheep; STHS, Small tailed-Han sheep; TAN, Tan sheep

Whole-genome resequencing data were collected from 108 sheep including three dairy (*n* = 33) and six non-dairy (*n* = 75) breeds and 200 goats including nine dairy (*n* = 147) and five non-dairy breeds (*n* = 53). All data were downloaded from three public databases: NCBI (https://www.ncbi.nlm.nih.gov/), EBI (https://www.ebi.ac.uk/) and NGDC (https://ngdc.cncb.ac.cn/?lang=zh). The geographic distributions of the sampled goat and sheep breeds are illustrated in Fig. 1A, and detailed sample information is provided in Additional file 1: Table S1. The latest reference genomes including *ARS-UI_Ramb_v3.0* for sheep and *ARS1.2* for goats were acquired from NCBI database. Fastq files for all individuals were efficiently retrieved using the IBM Aspera connect (https://www.ibm.com/products/aspera) followed by quality control processing with fastp (v0.23.4) software [20] to ensure data reliability.

### SNP calling and quality control

The cleaned sequencing reads were mapped to their respective reference genomes using BWA (v0.7.17) [21] and the resulting alignments were processed with SAMtools (v1.17) [22]. Variant calling was performed using GATK (v4.1.7.0) [23]. The raw variants were then filtered with VCFtools (v0.1.16) [24] by applying a minimum sequencing depth of 2 and retaining only biallelic sites, resulting in an intermediate VCF file. Finally, quality control was carried out using PLINK (v1.90) [25], with parameters set to a minor allele frequency (MAF) threshold of 0.01, a maximum missingness rate per genotype and individual of 0.1, and allowance for non-standard chromosome identifiers, to produce the final high-quality VCF file for downstream analysis.

### Population structure

Principal component analysis (PCA) was performed using PLINK (v1.90) [25], and the results were visualized in R (v4.4.1) [26]. To further elucidate population relationships, FastTree (v2.1.11) was employed to construct the phylogenetic tree [27]. Additionally, genetic structure was inferred using ADMIXTURE (v1.3.0) [28], which employs a maximum-likelihood framework to estimate ancestry components based on allele frequencies. *K* values ranging from 2 to 7 were tested to evaluate potential population clustering patterns.

### Genetic diversity

The genomic diversity was evaluated for dairy and non-dairy populations in sheep and goats, respectively. These parameters, including the observed heterozygosity (*Ho*), expected heterozygosity (*He*), minor allele frequencies (MAF), runs of homozygosity (ROH) were calculated via the PLINK software (v1.09) [25]. The linkage disequilibrium (LD) decay rate between pairs of autosomal SNPs was investigated with the *r*^2^ estimate using the PopLDecay (v3.42) software [29]. In addition, Tajima's D values were calculated in 50-kb windows using VCFtools (v0.1.16) [24] and the results were visualized in R (v4.4.1) [26].

### Selection signature analysis

Pairwise fixation index (*F*_ST_) and nucleotide diversity (*π*) ratio were applied as indicators to capture signals of selective sweeps related to milk production traits. The VCF tools (v0.1.16) [24] were used to identify genomic regions under potential selection. The *F*_ST_ was calculated between dairy and non-dairy populations using a sliding-window approach (50 kb window size, 25 kb step). *π* ratio was computed for each population in the same way. For sheep, the top 5% of both *F*_ST_ and *π* ratio values were defined as candidate selective signals, while for goats the top 1% thresholds were applied. We then focused on overlapping windows supported by both indices, and further identified common selective regions shared between sheep and goats. The overlapping SNPs located within these candidate regions were visualized using the VennDiagram package in R (v4.4.1) [26]. Subsequently, the candidate regions were annotated with bedtools (v2.31.1) [30] using the reference gene annotation files (sheep: *ARS-UI_Ramb_v3.0*; goats: *ARS1.2*).

### GO enrichment and KEGG pathway analyses

Gene Ontology (GO) and KEGG pathway enrichment analyses for the identified candidate genes were performed on DAVID website (https://david.ncifcrf.gov/) [31] with all annotated genes in the sheep and goat reference genomes serving as the background set. Significantly enriched terms and pathways were defined using a threshold of *P* < 0.05 and requiring at least two input genes per category.

### Haplotype analysis

The haplotype of key genes associated with milk production traits was analysed by first extracting their genomic regions from the VCF files using BCFtools (v1.18) [22], followed by compression with bgzip and indexing with tabix. Subsequently, genotype visualization and haplotype block comparisons between dairy and non-dairy populations in sheep and goats were performed using the GenotypeShow.pl script from the RectChr (v1.37) (https://github.com/hewm2008/RectChr) in a Linux environment.

### Transcriptome analysis

Public RNA-seq data (Additional file 1: Table S12) from the mammary glands of dairy goats and from ewes of dairy and non-dairy sheep breeds with varying milk production were analysed to assess the expression of these targeted genes. Raw paired-end reads were quality-trimmed and filtered using fastp (v0.23.4) [20]. Adapter sequences were automatically detected and removed (–detect_adapter_for_pe). Low-quality bases were trimmed from both ends (–cut_front, –cut_tail) using a sliding window of 4 bases and a mean quality threshold of Q20 (–cut_window_size 4, –cut_mean_quality 20). Reads containing more than five ambiguous bases (–n_base_limit 5) or shorter than 50 bp after trimming (–length_required 50) were discarded. PolyG and PolyX tails were also trimmed (–trim_poly_g, –trim_poly_x). The high-quality reads were then aligned to their respective reference genomes (sheep: *ARS-UI_Ramb_v3.0*; goats: *ARS1.2*) using STAR (v2.7.11b) [32], with default settings except for several specified options to improve alignment accuracy (–outFilterMismatchNmax 3, –outFilterMultimapNmax 10, –outSAMtype BAM SortedByCoordinate, and –quantMode TranscriptomeSAM). Properly paired alignments were retained, and the resulting sorted BAM files were generated using SAMtools (v1.17) [22]. Gene expression was quantified with featureCounts (v2.1.1) [33] with exon-level summarization (-t exon, -g gene_id) and paired-end counting enabled where applicable (-p). Differential expression analysis was performed using DESeq2 (v1.40.2) [34]. Prior to model fitting, the analyses were restricted to annotated protein-coding genes, and lowly expressed genes were removed by retaining only those with at least 10 counts in at least 50% of the samples (implemented as keep <-rowSums(counts(dds0) ≥ 10) ≥ (0.5 × ncol(dds0))), ensuring a robust set of reliably expressed transcripts for downstream modelling. To stabilize log_2_ fold-change (log_2_FC) estimates, DESeq2 effect-size shrinkage was applied using the lfcShrink function with the apeglm method [35], which reduced variance and prevented inflation of fold changes for genes with low or marginal expression. Differentially expressed genes were identified applying a threshold of |log_2_FC| > 1 and an adjusted *P* value < 0.05, following standard workflows. All visualizations were conducted in R (v4.4.1) [26].

## Results

### SNP discovery and characterization

The genomic dataset represented the most comprehensive collection for dairy-type sheep and goats, including 180 dairy samples (33 sheep and 147 goats) and 128 non-dairy samples (75 sheep and 53 goats), with an average sequencing depth of ~ 23 × across all samples (Additional file 1: Table S1). A total of 28,651,586 and 27,134,793 SNPs were identified in goats and sheep compared to the reference genome, respectively (Additional file 1: Table S2, Additional file 2: Fig. S1). In terms of SNP distribution, the non-coding transcript variants had the highest number of SNPs (28,627,648); while 28,319,838 SNPs were in intron; 17,771,888 SNPs were in intergenic regions; and 560,878 SNPs were in exon in goats. In sheep, SNPs were most abundant in the non-coding transcript regions with 60,542,026. The intronic regions contained 59,804,438 SNPs; intergenic regions 15,274,728 SNPs; while 1,316,837 variants were in exon.

### Genetic diversity and population structure

**Fig. 2:**
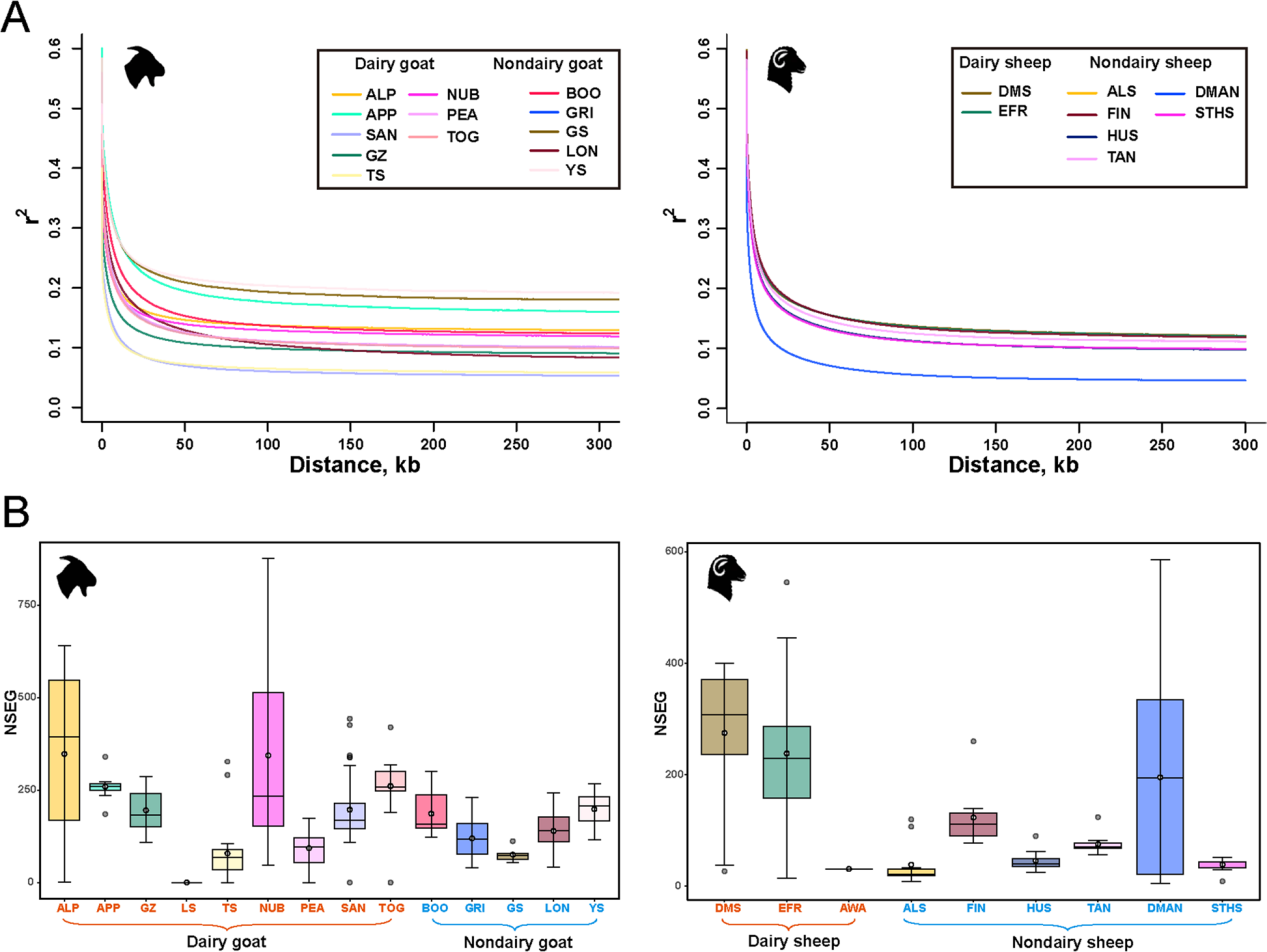
Genetic diversity analyses of goats and sheep. **A** Average LD decay in goats and sheep. **B** Distribution of the number of runs of homozygosity segments (NSEG) across goat and sheep breeds

In this study, the population genetic diversity of 14 different goat breeds (*n* = 200), and 9 sheep breeds (*n* = 108) was analysed. The mean values of *Ho*, *He*, and MAF in sheep and goats were displayed in Additional file 1: Tables S3 and S4. The *Ho* of the goat breeds ranged between 0.142–0.401, while *He* varied between 0.148–0.224 (Additional file 1: Table S3). Overall, majority of dairy breeds showed moderate levels of heterozygosity (*Ho* = 0.16–0.18; *He* = 0.15–0.19). The *Ho* among sheep breeds was between 0.270–1.000, while *He* varied between 0.297–0.500, and most breeds showed moderate and comparable diversity values (Additional file 1: Table S4). Additionally, LD decay, ROH segment count (NSEG) and Tajima’s D tests were also conducted. From the perspective of LD decay analysis (Fig. 2A), dairy breeds of both sheep and goats generally exhibited faster to moderate LD decay, consistent with higher recombination rates and greater genetic diversity. Number of ROH segment analysis (Fig. 2B) revealed higher inbreeding levels in dairy goat (e.g., ALP, NUB) and sheep breeds (e.g., DMS, EFR), while non-dairy breeds exhibited lower segment numbers, reflecting variability in genomic homozygosity across populations. In the tested goat and sheep populations, Tajima’s D values (Additional file 5: Fig. S4) around 0 indicated neutrality and demographic stability, while positive values suggested balancing selection and reduced rare alleles. In contrast, negative values observed in some breeds might reflect directional selection or recent expansion linked to intensive selection for dairy traits.

Based on the PCA results, the dairy and non-dairy goat breeds showed some mixed samples (Fig. 1B), with overlapping distributions along both PC1 and PC2, indicating a shared genetic background and possible historical crossbreeding. In contrast, dairy and non-dairy sheep breeds exhibited a pronounced genetic differentiation between these two groups (Fig. 1B). In addition, the PCA plots illustrated the geographic origin and breed-specific clustering of sheep and goats (Additional file 3: Fig. S2), and highlighted that most of European dairy goat breeds appear to overlap, suggesting shared ancestry or gene flow. The results of ADMIXTURE analysis on the genomes of both sheep and goat groups were given in Fig. 1C and Additional file 4: Fig. S3. At *K* = 6 (the optimal CV value), most dairy goat breeds (e.g., NUB, ALP, APP, GZ, TOG, SAN) exhibited mixed ancestral components, indicating extensive gene flow and admixture among dairy populations. In sheep, when *K* = 3, dairy breeds (e.g., AWA, DMS, EFR) remained closely related and shared a substantial proportion of common ancestry, consistent with their long history of selection for milk production. In contrast, non-dairy breeds showed a representation of cluster with minor contributions from other genetic backgrounds. Moreover, based on samples of whole genome SNP data, a phylogenetic tree was constructed for sheep and goats (Fig. 1D). The genetic similarity observed among some dairy goat breeds suggested a shared breeding history or common selection pressures for dairy traits. In contrast, the phylogenetic tree revealed distinct genetic groupings among sheep breeds, with clear separation between dairy and non-dairy types, reflecting their divergent selection histories and breeding objectives. These results consistently distinguished between dairy and non-dairy breeds in sheep and goats, revealing a clear separation via PCA, ADMIXTURE, and phylogenetic tree analyses, however, certain populations displayed mixed genetic signals.

### Selective sweeps

**Fig. 3:**
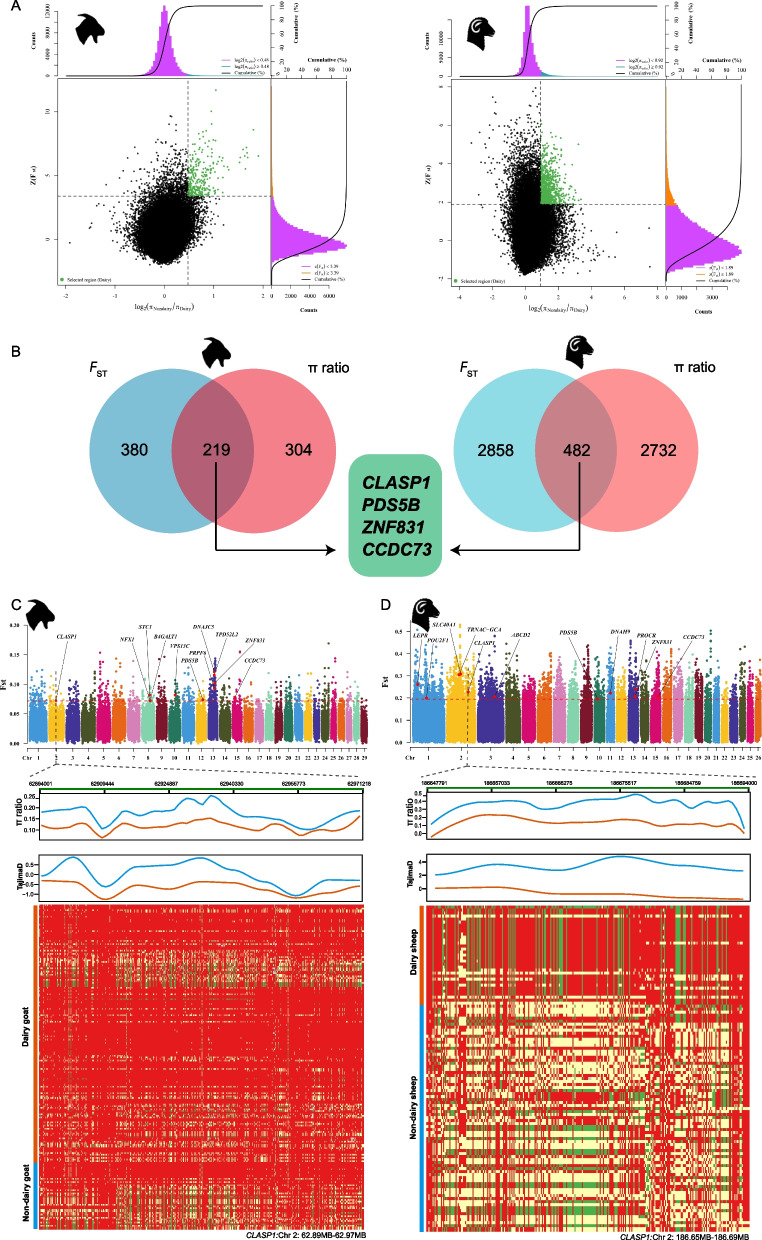
Selective signatures of goats and sheep by pairwise *F*_ST_ and π ratio methods. **A** Distribution of log_2_ (π ratios) and pairwise *F*_ST_ values calculated in 50-kb sliding windows in dairy and non-dairy goats and sheep. **B** Volcano plot showing genes identified in goats and sheep using combined pairwise *F*_ST_ and π ratio approaches. **C** Manhattan plot (*F*_ST_) and selection signature metrics (π ratio and Tajima’s D) for the *CLASP1* gene in goats. **D** Manhattan plot (*F*_ST_) and selection signature metrics (π ratio and Tajima’s D) for the *CLASP1* gene in sheep

In this study, the pairwise *F*_ST_ and π ratio analyses were employed to robustly identify genomic variants associated with milk traits in the studied sheep and goat populations. The top 1% windows of the goat population differentiation index were extracted, and 380 genes were annotated in goats by pairwise *F*_ST_ method (Fig. 3, Additional file 1: Table S5); while in sheep, 2,858 candidate genes were screened based on the top 5% of pairwise* F*_ST_ values (Fig. 3, Additional file 1: Table S8). In addition, using the π ratio method, 304 candidate genes were identified in goats from the top 1% windows (Additional file 1: Table S6) and 2,732 candidate genes were detected in sheep from the top 5% windows (Additional file 1: Table S9).

Non-dairy goats were used as the background and dairy goats as the selected population, identifying 219 candidate genes within the top 1% of genomic regions based on combined *F*_ST_ and π ratio analyses (Fig. 3B, Additional file 1: Table S7). Candidate genes such as *B4GALT1, PRPF6*, *VPS13C*, *TPD52L2*, *NFX1, CLASP1, STC1, PRPF6, DNAJC5* were found to be related to milk traits [36–40]. The results of enrichment analysis (Additional file 6: Fig. S5) showed that these candidate genes were enriched to nuclear chromosome segregation (GO: 0098813, *P* = 0.011618), positive regulation of mitotic cell cycle G1/S transition (GO: 1900087, *P* = 0.028604), regulation of protein metabolic process (GO: 0051246, *P* = 0.012634), U2-type spliceosomal complex (GO: 0005684, *P* = 0.006976). KEGG was enriched in pathways such as the endocrine resistance (chx01522, *P* = 0.015184), propanoate metabolism (chx00640, *P* = 0.037743), renin secretion (chx04924, *P* = 0.035922) (Additional file 1: Table S11).

Potential selection regions were identified by calculating the top 5% of *F*_ST_ and π ratio between non-dairy and dairy sheep populations, and the overlapping genes between the two methods were 482 (Fig. 3B; Additional file 1: Table S10). In sheep, the *TRNAC-GCA*, *ACACA*, *POU2F1*, *LEPR*, *ABCD2*, *CLASP1*, *SLC40A1*, *DNAH9*, *PROCR* genes were detected to be involved in the regulation of milk production traits [41–45]. Moreover, the genes were enriched for Wnt signaling pathway (GO: 0016055, *P* = 0.001059), multicellular organism development (GO: 0007275 *P* = 0.001783), regulation of metabolic process (GO: 0019222, *P* = 0.002825), fatty acid oxidation (GO: 0019395, *P* = 0.013286), AMP metabolic process (GO:0046033, *P* = 0.038105) (Additional file 6: Fig. S5). In addition, KEGG was enriched in beta-Alanine metabolism (oas00410, *P* = 5.15 × 10^–4^), Hippo signaling pathway (oas04392, *P* = 0.018137), ECM-receptor interaction pathways (oas04512, *P* = 0.029082) (Additional file 1: Table S11).

### Shared selective signatures of milk traits in sheep and goats

By combining *F*_ST_ and π ratio analyses, genes such as *CLASP1*, *PDS5B*, *ZNF831*, and *CCDC73* were commonly identified in both sheep and goats (Fig. 3). Further, haplotype structure analysis of selected genes was conducted in order to better understand the genetic architecture underlying milk traits. In the *CLASP1* gene region, dairy goats displayed lower π ratio and Tajima’s D values than non-dairy goats, suggesting that this gene had likely undergone recent positive selection for milk traits (Fig. 3C). In the orthologous region in sheep, dairy sheep also exhibited reduced π ratio and Tajima’s D values compared to non-dairy sheep, indicating a convergent selection pressure on this region across both species (Fig. 3D). Besides, haplotype analysis of the *CLASP1* gene revealed distinct genotype patterns between dairy and non-dairy breeds in both sheep and goats. Notably, dairy breeds exhibited more conserved haplotype blocks in several regions, indicating potential selective pressure associated with milk production traits. In addition, the haplotype structures of other candidate genes were given in Additional file 7: Fig. S6.

### Comparative analysis of gene expression between dairy and non-dairy breeds in sheep and goats

**Fig. 4:**
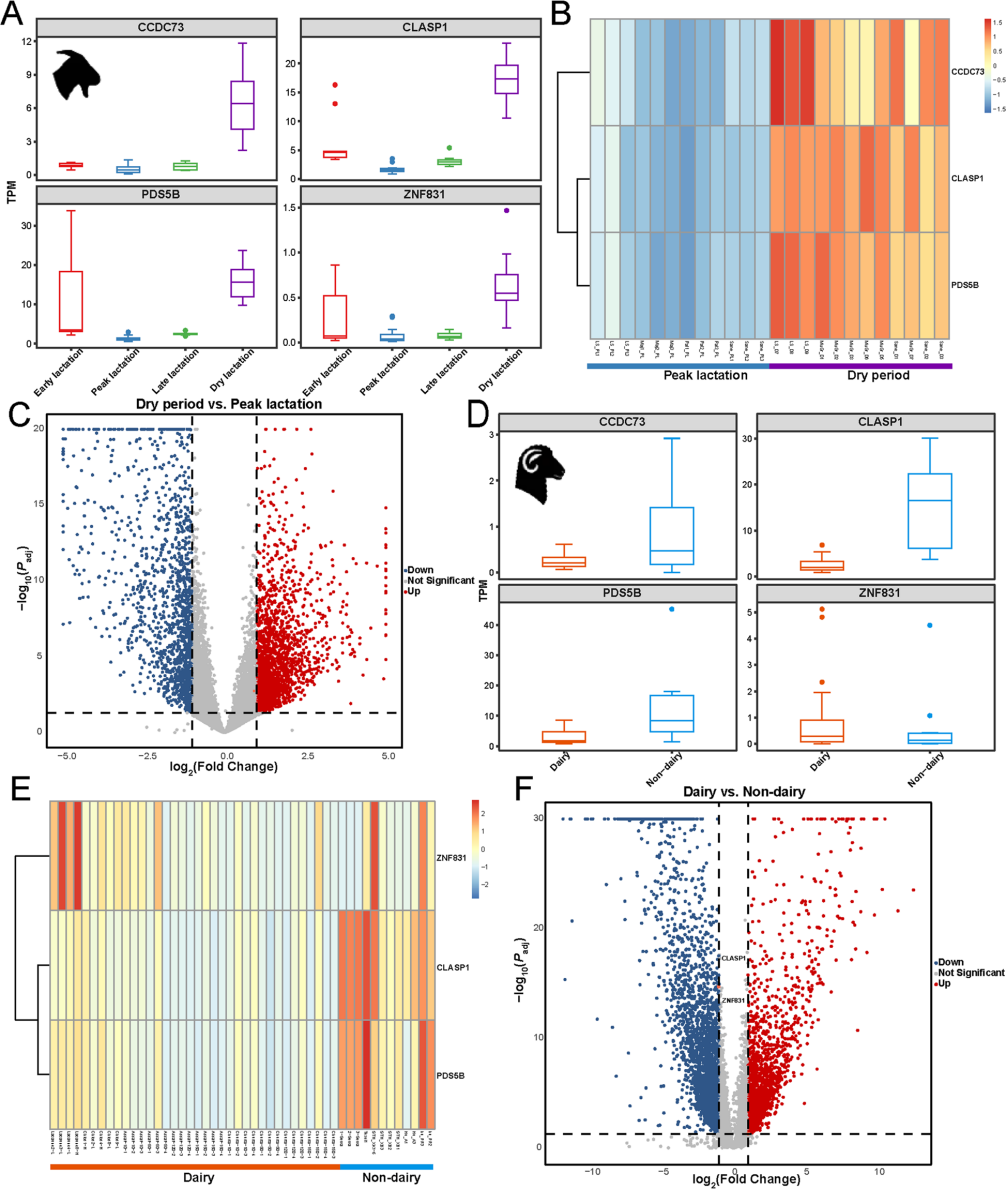
Gene expression analysis of candidate genes in goats and sheep. **A** Boxplot showing the TPM (transcripts per million) distribution of candidate genes in goat samples during different lactation stages. **B** Heatmap of the DEGs (differentially expressed genes) cluster analysis between the dry period and peak lactation in goats. **C** Volcano plot of DEGs between the dry period and peak lactation in goats. **D** Boxplot showing the TPM distribution of candidate genes in dairy and non-dairy sheep breeds. **E** Heatmap of the DEGs cluster analysis between dairy and non-dairy sheep breeds. **F** Volcano plot of DEGs between dairy and non-dairy sheep breeds

To further explore candidate gene expression in the mammary gland, differential expression analysis revealed clear phase-specific expression patterns of these genes in dairy goats (Fig. 4A, Additional file 8: Fig. S7). The analysis demonstrated that *CLASP1* was significantly upregulated (*P*_adj_ = 0.033) during dry-off stage compared to the peak lactation period (Fig. 4, Additional file 1: Table S15); while its expression was also elevated at early lactation (*P*_adj_ = 0.005) compared to the late lactation stage (Additional file 8: Fig. S7A), suggesting its role in the initiation and recovery phases in lactation. Meanwhile, the *PDS5B* gene was markedly upregulated in early versus peak lactation (*P*_adj_ = 1.87 × 10⁻^7^) and early versus late lactation (*P*_adj_ = 6.15 × 10⁻^5^), supporting its involvement in mammary tissue remodeling (Additional file 1: Tables S16 and S17, Additional file 8: Fig. S7A–D). The *CCDC73* gene showed consistently low expression throughout lactation but was significantly upregulated in the dry period compared with peak lactation (*P*_adj_ = 0.002), while *ZNF831* was elevated in early lactation compared with late lactation (*P*_adj_ = 0.031) (Additional file 8: Fig. S7C). Collectively, these patterns highlighted that the studied genes predominantly contributed to lactation stage transitions, with *CLASP1* and *PDS5B* linked to early and recovery phases, *CCDC73* associated with involution, and *ZNF831* with early lactation activation.

In sheep, differential expression analysis between dairy and non-dairy breeds revealed noticeable transcriptional profiles (Fig. 4D). In particular, *CLASP1* showed significant differences between breeds, with strong downregulation in dairy sheep (*P*_adj_ = 1.92 × 10^−^^15^) compared to non-dairy sheep breeds, likely reflecting selective pressures for milk production (Fig. 4E and F, Additional file 1: Table S20). Similarly, *PDS5B* was also downregulated in dairy sheep (*P*_adj_ = 3.26 × 10^−^^5^), further supporting the presence of breed-specific regulatory patterns. By contrast, *ZNF831* exhibited a tendency toward higher expression (*P*_adj_ = 0.048) in dairy breeds, indicating a possible role in regulatory processes (Fig. 4E and F). These patterns highlighted fundamental transcriptional differences between dairy and non-dairy sheep breeds, suggesting that enhanced milk production in sheep was accompanied by suppression of broader tissue remodeling genes. Thus, the *CLASP1* gene could be considered as a target of convergent selection for milk traits in both sheep and goats.

## Discussion

With the emergence of climatic extremes and the increasing impact of human activities, the pressure on the survival of dairy sheep and goat populations is increasing [46]. Comprehensive understanding of both the genetic structure and functional genes in both species can provide guidance for population management and conservation of genetic resources, and help to develop more effective breeding strategies [47, 48]. Despite numerous studies on the genetic architecture of milk production in sheep and goats, the key genes shaped by convergent selection and responsible for major phenotypic variation remain poorly defined. Here, we integrated genomic and transcriptomic data from dairy and non-dairy breeds of sheep and goats to examine whether regions under selection are shared between these two species. Population structure analysis revealed distinct genetic separation between dairy and non-dairy sheep, while goat populations were more admixed. This pattern is likely due to the long history of crossbreeding and selection in dairy goats, which has led to shared genetic components across populations and is consistent with prior comparative analyses [47, 49, 50].

Notably, the SNP-based selective sweep analyses revealed convergent signatures of selection associated with milk traits in both sheep and goat genomes, highlighting molecular parallelism at four shared genes (*CLASP1*, *PDS5B*, *ZNF831*, *CCDC73*). To elucidate their biological relevance, the haplotype analysis was performed, which showed that dairy breeds in both species exhibited more conserved haplotype blocks and lower nucleotide diversity. These consistent diversity patterns provide strong evidence for similar selective pressures on these genomic regions. Interestingly, transcriptome data analysis showed that the expression of these genes are closely associated with lactation transitions and mammary tissue remodeling in goats. The contrasting expression patterns observed between dairy and non-dairy sheep breeds pointed to selective pressures shaping transcriptional regulation related to milk production. Notably, two of these genes, *CLASP1* and *ZNF831* were previously identified as candidate genes for milk traits [51–53], confirming their role in lactation-related functions. For example, CLASP1, a member of microtubule plus-end–tracking proteins, regulates focal adhesion turnover by coupling microtubule organization with extracellular matrix remodeling and vesicle transport, thereby facilitating coordinated cell migration [54]. ZNF831 belongs to the major family of transcription factors, the zinc finger proteins, which are characterized by zinc finger motifs and are implicated in the regulation of cell apoptosis [55]. Additionally, PDS5B, one of the two vertebrate paralogs of the cohesin-associated protein PDS5, plays both redundant and unique roles in regulating cohesin-mediated processes, including sister chromatid cohesion, DNA repair, transcription, and replication [56]. *CCDC73*, a member of the coiled-coil domain–containing (CCDC) gene family, encodes proteins implicated in intercellular signal transduction and transcriptional regulation, and alterations in CCDC family genes have been associated with diverse pathological processes, such as tumorigenesis [57]. These results demonstrated molecular parallelism, where similar selective pressures for dairy traits had independently shaped genetic variation at the same loci in both species, highlighting shared genetic mechanisms driving convergent phenotypic evolution in domesticated ruminants.

The identification of a limited set of convergent genes in this study might reflect two constraints: (1) the relatively small cohort of dairy sheep, due to sample availability, and (2) the reliance on SNP data rather than structural variants. Future work with larger sample sizes and diverse genomic data will provide a more comprehensive view of convergent evolution for milk traits. Nevertheless, our findings establish a foundational link between selection signals and mammary gland biology. Specifically, the role of *CLASP1* suggests it contributes to the cellular remodeling essential for mammary gland function across physiological stages. These outcomes provide a new direction for unraveling the genetic basis of milk production in small ruminants, with potential applications for advancing dairy breeding programs.

## Conclusions

This study identified genes (e.g., *CLASP1*) under convergent selection associated with milk traits in both sheep and goats, revealing new targets for genetic enhancement of milk production in small ruminants. Complementary transcriptome data revealed distinct expression patterns, suggesting roles in lactation and breed-specific traits. These findings will provide insights into the understanding of the genetic mechanisms underlying convergent evolution in dairy traits, and provide valuable insights for future breeding strategies aimed at improving milk production in sheep and goats.

## Supplementary Information

Additional file 1: Table S1. WGS data information. Table S2. SNP information. Table S3. Statistics on diversity parameters of different goat breeds. Table S4. Statistics on diversity parameters of different sheep breeds. Table S5. The top 1% of candidate genes under selection in goats by *F*_ST_. Table S6. The top 1% of candidate genes under selection in goats by π ratio. Table S7. Overlapping selection candidate genes in goats by *F*_ST_ and π ratio. Table S8. The top 5% of candidate genes under selection in sheep by *F*_ST_. Table S9. The top 5% of candidate genes under selection in sheep by π ratio. Table S10. Overlapping selection candidate genes in sheep by *F*_ST_ and π ratio. Table S11. KEGG pathway enrichment of candidate genes selected by *F*_ST_ and π ratio in goats and sheep. Table S12. Description of RNA-seq datasets in goats and sheep. Table S13. Gene expression count matrix of goat RNA-seq data. Table S14. Gene expression TPM Matrix of goat RNA-seq data. Table S15. Goat DESeq2 result of dry period vs. peak lactation. Table S16. Goat DESeq2 result of early vs. late lactation. Table S17. Goat DESeq2 result of early vs. peak lactation. Table S18. Gene expression count matrix of sheep RNA-seq data. Table S19. Gene expression TPM matrix of sheep RNA-seq data. Table S20. Sheep DESeq2 result of dairy vs. non-dairy.

Additional file 2: Fig. S1. Characterization of SNP variants in goats and sheep. **A** Distribution of SNPs within 1 Mb sliding windows across goat and sheep autosomes. **B** SNP number distribution across different genomic regions in goats and sheep.

Additional file 3: Fig. S2. Principal component analysis (PCA) of goat and sheep breeds. **A** PCA of different goat breeds. **B** PCA of goat populations grouped by geographic origin (Asia, Europe, Oceania). **C** PCA of different sheep breeds. **D** PCA of sheep populations grouped by geographic origin (Asia, Africa, Europe, Oceania). **E** PCA of dairy and non-dairy goat and sheep populations.

Additional file 4: Fig. S3. Population structure analysis of dairy and non-dairy goats and sheep. **A **ADMIXTURE analysis of dairy and non-dairy goats and sheep at *K* = 2–7. **B **Cross-validation error values for different *K* clusters in goats and sheep.

Additional file 5: Fig. S4. Tajima’s D values in different goat and sheep breeds.

Additional file 6: Fig. S5. Gene Ontology (GO) enrichment analysis of goats and sheep.

Additional file 7: Fig. S6. Haplotype block structures of candidate genes in dairy and non-dairy breeds. **A** Haplotype blocks of *PDS5B* in goats and sheep. **B** Haplotype blocks of *ZNF831 *in goats and sheep. **C** Haplotype blocks of *CCDC73 *in goats and sheep.

Additional file 8: Fig. S7. Transcriptomic analysis of goat samples during different lactation stages. **A** Heatmap of candidate gene expressions between early and late lactation in goats. **B** Heatmap of candidate gene expressions in early vs. peak lactation in goats. **C** Volcano plot of DEGs between early and late lactation in goats. **D** Volcano plot of DEGs between early and peak lactation in goats.

## Data Availability

The whole genome re-sequencing and RNA-sequencing data used for the study are publicly available under the sample accession numbers listed in Additional file 1: Table S1 and Table S12, respectively. All scripts used for this work were performed using open-source software tools and are available from the corresponding authors upon request.

## Abbreviations

*F*_ST_: Fixation index
GWAS: Genome-wide association study
MAF: Minor allele frequency
PCA: Principal component analysis
ROH: Runs of homozygosity
SNP: Single nucleotide polymorphism
WGS: Whole genome sequencing

## References

[1] Witt KE, Huerta-Sánchez E. Convergent evolution in human and domesticate adaptation to high-altitude environments. Philos Trans R Soc Lond B Biol Sci. 2019;374(1777):20180235. 10.1098/rstb.2018.0235. 31154977 10.1098/rstb.2018.0235 PMC6560271

[2] Wu DD, Yang CP, Wang MS, Dong KZ, Yan DW, Hao ZQ, et al. Convergent genomic signatures of high-altitude adaptation among domestic mammals. Natl Sci Rev. 2020;7(6):952–63. 10.1093/nsr/nwz213. 34692117 10.1093/nsr/nwz213 PMC8288980

[3] Liang D, Zhao P, Si J, Fang L, Pairo-Castineira E, Hu X, et al. Genomic analysis revealed a convergent evolution of LINE-1 in coat color: a case study in water buffaloes (*Bubalus bubalis*). Mol Biol Evol. 2021;38(3):1122–36. 10.1093/molbev/msaa279. 33212507 10.1093/molbev/msaa279 PMC7947781

[4] Jiang L, Kon T, Chen C, Ichikawa R, Zheng Q, Pei L, et al. Whole-genome sequencing of endangered Zhoushan cattle suggests its origin and the association of MC1R with black coat colour. Sci Rep. 2021;11:17359. 10.1038/s41598-021-96896-2. 10.1038/s41598-021-96896-2 PMC8405626 34462508

[5] Dutta P, Talenti A, Young R, Jayaraman S, Callaby R, Jadhav SK, et al. Whole genome analysis of water buffalo and global cattle breeds highlights convergent signatures of domestication. Nat Commun. 2020;11:4739. 10.1038/s41467-020-18550-1. 10.1038/s41467-020-18550-1 PMC7505982 32958756

[6] Lv FH, Cao YH, Liu GJ, Luo LY, Lu R, Liu MJ, et al. Whole-genome resequencing of worldwide wild and domestic sheep elucidates genetic diversity, introgression, and agronomically important loci. Mol Biol Evol. 2022;39(2):msab353. 10.1093/molbev/msab353. 34893856 10.1093/molbev/msab353 PMC8826587

[7] Akhatayeva Z, Dan H, Salehian-Dehkordi H, Seiteuov T, Abdurasulov A, Aitjanov R, et al. Recent advances in genomic studies for domestication and genetic improvement of traits in goats. J Integr Agric. In press. 10.1016/j.jia.2025.07.020.

[8] Stiner MC, Munro ND, Buitenhuis H, Duru G, Özbaşaran M. An endemic pathway to sheep and goat domestication at Aşıklı Höyük (Central Anatolia, Turkey). Proc Natl Acad Sci U S A. 2022;25(4):e2110930119. 10.1073/pnas.2110930119. 10.1073/pnas.2110930119 PMC8795544 35042793

[9] Alberto FJ, Boyer F, Orozco-terWengel P, Streeter I, Servin B, de Villemereuil P, et al. Convergent genomic signatures of domestication in sheep and goats. Nat Commun. 2018;9(1):813. 10.1038/s41467-018-03206-y. 29511174 10.1038/s41467-018-03206-y PMC5840369

[10] Li M, Dehkordi HS. Domestic and wild sheep origins, genetics and breeding. London and Cambridge: Academic Press; 2026.

[11] Xu S, Akhatayeva Z, Liu J, Feng X, Yu Y, Badaoui B, et al. Genetic advancements and future directions in ruminant livestock breeding: from reference genomes to multiomics innovations. Sci China Life Sci. 2025;68(4):934–60. 10.1007/s11427-024-2744-4. 10.1007/s11427-024-2744-4 39609363

[12] Yang J, Wang DF, Huang JH, Zhu QH, Luo LY, Lu R, et al. Structural variant landscapes reveal convergent signatures of evolution in sheep and goats. Genome Biol. 2024;25:148. 10.1186/s13059-024-03288-6. 10.1186/s13059-024-03288-6 PMC11155191 38845023

[13] Khan MZ, Ma Y, Ma J, Xiao J, Liu Y, Liu S, et al. Association of DGAT1 with cattle, buffalo, goat, and sheep milk and meat production traits. Front Vet Sci. 2021;8:712470. 10.3389/fvets.2021.712470. 34485439 10.3389/fvets.2021.712470 PMC8415568

[14] Jiang L, Liu X, Yang J, Wang H, Jiang J, Liu L, et al. Targeted resequencing of GWAS loci reveals novel genetic variants for milk production traits. BMC Genomics. 2014;15:1105. 10.1186/1471-2164-15-1105. 25510969 10.1186/1471-2164-15-1105 PMC4377845

[15] Huang Z, Tang Y, Zhou J, Xu D, Lin X, Cheng M, et al. Genome-wide association study on dairy goat milk production traits using three models. Front Genet. 2025;16:1650836. 10.3389/fgene.2025.1650836. 40919429 10.3389/fgene.2025.1650836 PMC12411178

[16] Rezvannejad E, Asadollahpour Nanaei H, Esmailizadeh A. Detection of candidate genes affecting milk production traits in sheep using whole-genome sequencing analysis. Vet Med Sci. 2022;8(3):1197–204. 10.1002/vms3.731. 35014209 10.1002/vms3.731 PMC9122411

[17] Saleh AA, Rashad AM, Hassanine NN, Sharaby MA. Candidate genes and signature of selection associated with different biological aspects and general characteristics of goat. Emerg Anim Species. 2022;5:100013. 10.1016/j.eas.2022.100013.

[18] Suárez-Vega A, Gutiérrez-Gil B, Arranz JJ. Transcriptome expression analysis of candidate milk genes affecting cheese-related traits in 2 sheep breeds. J Dairy Sci. 2016;99(8):6381–90. 10.3168/jds.2016-11048. 27179853 10.3168/jds.2016-11048

[19] Guan D, Landi V, Luigi-Sierra MG, Delgado JV, Such X, Castelló A, et al. Analyzing the genomic and transcriptomic architecture of milk traits in Murciano-Granadina goats. J Anim Sci Biotechnol. 2020;11:35. 10.1186/s40104-020-00435-4. 32175082 10.1186/s40104-020-00435-4 PMC7065321

[20] Chen S, Zhou Y, Chen Y, Gu J. Fastp: an ultra-fast all-in-one FASTQ preprocessor. Bioinformatics. 2018;34(17):i884–90. 10.1093/bioinformatics/bty560. 30423086 10.1093/bioinformatics/bty560 PMC6129281

[21] Li H, Durbin R. Fast and accurate short read alignment with Burrows-Wheeler transform. Bioinformatics. 2009;25(14):1754–60. 10.1093/bioinformatics/btp324. 19451168 10.1093/bioinformatics/btp324 PMC2705234

[22] Danecek P, Bonfield JK, Liddle J, Marshall J, Ohan V, Pollard MO, et al. Twelve years of SAMtools and BCFtools. Gigascience. 2021;10(2):giab008. 10.1093/gigascience/giab008. 33590861 10.1093/gigascience/giab008 PMC7931819

[23] McKenna A, Hanna M, Banks E, Sivachenko A, Cibulskis K, Kernytsky A, et al. The genome analysis toolkit: a MapReduce framework for analyzing next-generation DNA sequencing data. Genome Res. 2010;20(9):1297–303. 10.1101/gr.107524.110. 20644199 10.1101/gr.107524.110 PMC2928508

[24] Danecek P, Auton A, Abecasis G, Albers CA, Banks E, DePristo MA, et al. 1000 genomes project analysis group. The variant call format and VCFtools. Bioinformatics. 2011;27(15):2156–8. 10.1093/bioinformatics/btr330. 21653522 10.1093/bioinformatics/btr330 PMC3137218

[25] Purcell S, Neale B, Todd-Brown K, Thomas L, Ferreira MA, Bender D, et al. PLINK: a tool set for whole-genome association and population-based linkage analyses. Am J Hum Genet. 2007;81(3):559–75. 10.1086/519795. 17701901 10.1086/519795 PMC1950838

[26] Ihaka R, Gentleman R. R: a language for data analysis and graphics. J Comput Graph Stat. 1996;5(3):299–314. 10.1080/10618600.1996.10474713.

[27] Price MN, Dehal PS, Arkin AP. Fasttree: computing large minimum evolution trees with profiles instead of a distance matrix. Mol Biol Evol. 2009;26(7):1641–50. 10.1093/molbev/msp077. 19377059 10.1093/molbev/msp077 PMC2693737

[28] Alexander DH, Novembre J, Lange K. Fast model-based estimation of ancestry in unrelated individuals. Genome Res. 2009;19(9):1655–64. 10.1101/gr.094052.109. 19648217 10.1101/gr.094052.109 PMC2752134

[29] Zhang C, Dong SS, Xu JY, He WM, Yang TL. PopLDdecay: a fast and effective tool for linkage disequilibrium decay analysis based on variant call format files. Bioinformatics. 2019;35(10):1786–8. 10.1093/bioinformatics/bty875. 30321304 10.1093/bioinformatics/bty875

[30] Quinlan AR, Hall IM. BEDtools: a flexible suite of utilities for comparing genomic features. Bioinformatics. 2010;26(6):841–2. 10.1093/bioinformatics/btq033. 20110278 10.1093/bioinformatics/btq033 PMC2832824

[31] Sherman BT, Hao M, Qiu J, Jiao X, Baseler MW, Lane HC, et al. DAVID: a web server for functional enrichment analysis and functional annotation of gene lists (2021 update). Nucleic Acids Res. 2022;50(W1):W216–21. 10.1093/nar/gkac194. 35325185 10.1093/nar/gkac194 PMC9252805

[32] Dobin A, Davis CA, Schlesinger F, Drenkow J, Zaleski C, Jha S, et al. STAR: ultrafast universal RNA-seq aligner. Bioinformatics. 2013;29(1):15–21. 10.1093/bioinformatics/bts635. 23104886 10.1093/bioinformatics/bts635 PMC3530905

[33] Liao Y, Smyth GK, Shi W. Featurecounts: an efficient general purpose program for assigning sequence reads to genomic features. Bioinformatics. 2013;30(7):923–30. 10.1093/bioinformatics/btt656. 24227677 10.1093/bioinformatics/btt656

[34] Love MI, Huber W, Anders S. Moderated estimation of fold change and dispersion for RNA-seq data with DESeq2. Genome Biol. 2014;15(12):550. 10.1186/s13059-014-0550-8. 25516281 10.1186/s13059-014-0550-8 PMC4302049

[35] Zhu A, Ibrahim JG, Love MI. Heavy-tailed prior distributions for sequence count data: removing the noise and preserving large differences. Bioinformatics. 2019;35(12):2084–92. 10.1093/bioinformatics/bty895. 30395178 10.1093/bioinformatics/bty895 PMC6581436

[36] Crisà A, Ferrè F, Chillemi G, Moioli B. RNA-Sequencing for profiling goat milk transcriptome in colostrum and mature milk. BMC Vet Res. 2016;12:264. 10.1186/s12917-016-0881-7. 10.1186/s12917-016-0881-7 PMC5123407 27884183

[37] Xiong J, Bao J, Hu W, Shang M, Zhang L. Whole-genome resequencing reveals genetic diversity and selection characteristics of dairy goat. Front Genet. 2023;13:1044017. 10.3389/fgene.2022.1044017. 10.3389/fgene.2022.1044017 PMC9852865 36685859

[38] Jiang J, Liu L, Gao Y, Shi L, Li Y, Liang W, et al. Determination of genetic associations between indels in 11 candidate genes and milk composition traits in Chinese Holstein population. BMC Genet. 2019;20:48. 10.1186/s12863-019-0751-y. 10.1186/s12863-019-0751-y PMC6537361 31138106

[39] Asadollahpour Nanaei H, Dehghani Qanatqestani M, Esmailizadeh A. Whole-genome resequencing reveals selection signatures associated with milk production traits in African Kenana dairy zebu cattle. Genomics. 2020;112(1):880–5. 10.1016/j.ygeno.2019.06.002. 10.1016/j.ygeno.2019.06.002 31170439

[40] Wu X, Lund MS, Sahana G, Guldbrandtsen B, Sun D, Zhang Q, et al. Association analysis for udder health based on SNP-panel and sequence data in Danish Holsteins. Genet Sel Evol. 2015;47:50. 10.1186/s12711-015-0129-1. 10.1186/s12711-015-0129-1 PMC4472403 26087655

[41] Moioli B, Scatà MC, De Matteis G, Annicchiarico G, Catillo G, Napolitano F. The ACACA gene is a potential candidate gene for fat content in sheep milk. Anim Genet. 2013;44(5):601–3. 10.1111/age.12036. 10.1111/age.12036 23488977

[42] Yang J, Jiang J, Liu X, Wang H, Guo G, Zhang Q, et al. Differential expression of genes in milk of dairy cattle during lactation. Anim Genet. 2016;47(2):174–80. 10.1111/age.12394. 10.1111/age.12394 PMC5064620 26692495

[43] Glantz M, Lindmark Månsson H, Stålhammar H, Paulsson M. Effect of polymorphisms in the leptin, leptin receptor, and acyl-coenzyme A:diacylglycerol acyltransferase 1 (DGAT1) genes and genetic polymorphism of milk proteins on cheese characteristics. J Dairy Sci. 2011;94(7):3295–304. 10.3168/jds.2011-4317. 10.3168/jds.2011-4317 21700014

[44] Zhou F, Yang Q, Lei C, Chen H, Lan X. Relationship between genetic variants of POU1F1, PROP1, IGFBP3 genes and milk performance in Guanzhong dairy goats. Small Rumin Res. 2016;140:40–5. 10.1016/j.smallrumres.2016.05.015.

[45] Fang M, Fu W, Jiang D, Zhang Q, Sun D, Ding X, et al. A multiple-SNP approach for genome-wide association study of milk production traits in Chinese Holstein cattle. PLoS One. 2014;9(8):e99544. 10.1371/journal.pone.0099544. 10.1371/journal.pone.0099544 PMC4141689 25148050

[46] Serranito B, Cavalazzi M, Vidal P, Taurisson-Mouret D, Ciani E, Bal M, et al. Local adaptations of Mediterranean sheep and goats through an integrative approach. Sci Rep. 2021;11:21363. 10.1038/s41598-021-00682-z. 10.1038/s41598-021-00682-z PMC8560853 34725398

[47] Amiri Ghanatsaman Z, Ayatolahi Mehrgardi A, Asadollahpour Nanaei H, Esmailizadeh A. Comparative genomic analysis uncovers candidate genes related with milk production and adaptive traits in goat breeds. Sci Rep. 2023;13:8722. 10.1038/s41598-023-35973-0. 10.1038/s41598-023-35973-0 PMC10229596 37253766

[48] Li R, Ma Y, Jiang L. Review: research progress of dairy sheep milk genes. Agriculture. 2022;12(2):169. 10.3390/agriculture12020169.

[49] Peng W, Zhang Y, Gao L, Wang S, Liu M, Sun E, et al. Investigation of selection signatures of dairy goats using whole-genome sequencing data. BMC Genomics. 2025;26:234. 10.1186/s12864-025-11437-9. 40069586 10.1186/s12864-025-11437-9 PMC11899394

[50] Li R, Zhao Y, Liang B, Pu Y, Jiang L, Ma Y. Genome-wide signal selection analysis revealing genes potentially related to sheep-milk-production traits. Animals. 2023;13(10):1654. 10.3390/ani13101654. 37238084 10.3390/ani13101654 PMC10215608

[51] Wang Z, Zhu B, Niu H, Zhang W, Xu L, Xu L, et al. Genome wide association study identifies SNPs associated with fatty acid composition in Chinese Wagyu cattle. J Anim Sci Biotechnol. 2019;10:27. 10.1186/s40104-019-0322-0. 30867906 10.1186/s40104-019-0322-0 PMC6399853

[52] Zhao J, Shi C, Kamalibieke J, Gong P, Mu Y, Zhu L, et al. Whole genome and transcriptome analyses in dairy goats identify genetic markers associated with high milk yield. Int J Biol Macromol. 2025;292:139192. 10.1016/j.ijbiomac.2024.139192. 39736302 10.1016/j.ijbiomac.2024.139192

[53] Sahana G, Guldbrandtsen B, Thomsen B, Holm LE, Panitz F, Brøndum RF, et al. Genome-wide association study using high-density single nucleotide polymorphism arrays and whole-genome sequences for clinical mastitis traits in dairy cattle. J Dairy Sci. 2014;97(11):7258–75. 10.3168/jds.2014-8141. 25151887 10.3168/jds.2014-8141

[54] Stehbens SJ, Paszek M, Pemble H, Ettinger A, Gierke S, Wittmann T. CLASPs link focal-adhesion-associated microtubule capture to localized exocytosis and adhesion site turnover. Nat Cell Biol. 2014;16(6):558–70. 10.1038/ncb2975. 10.1038/ncb2975 PMC4108447 24859005

[55] Fan J, Zhang Z, Chen H, Chen D, Yuan W, Li J, et al. Zinc finger protein 831 promotes apoptosis and enhances chemosensitivity in breast cancer by acting as a novel transcriptional repressor targeting the STAT3/Bcl2 signaling pathway. Genes Dis. 2022;11:430–48. 10.1016/j.gendis.2022.11.023. 37588209 10.1016/j.gendis.2022.11.023 PMC10425751

[56] Zhang N, Coutinho LE, Pati D. PDS5A and PDS5B in cohesin function and human disease. Int J Mol Sci. 2021;22(11):5868. 10.3390/ijms22115868. 34070827 10.3390/ijms22115868 PMC8198109

[57] Liu Z, Yan W, Liu S, Liu Z, Xu P, Fang W. Regulatory network and targeted interventions for CCDC family in tumor pathogenesis. Cancer Lett. 2023;565:216225. 10.1016/j.canlet.2023.216225. 37182638 10.1016/j.canlet.2023.216225

