# Supplementary figures and images for: Genomic analysis reveals convergent signatures of selection for milk traits in sheep and goats
Source: J Anim Sci Biotechnol. 2026 Feb 6;17:22. doi: 10.1186/s40104-025-01334-2 (PMC12879437; doi:10.1186/s40104-025-01334-2)

A

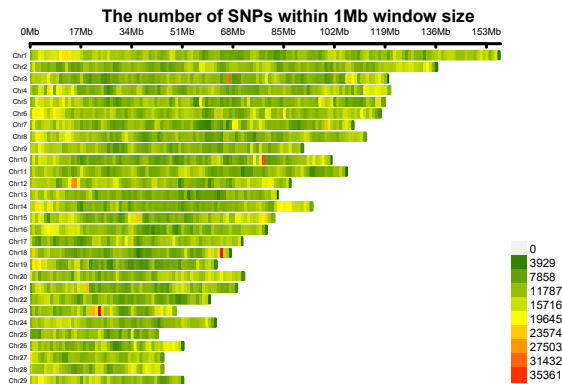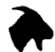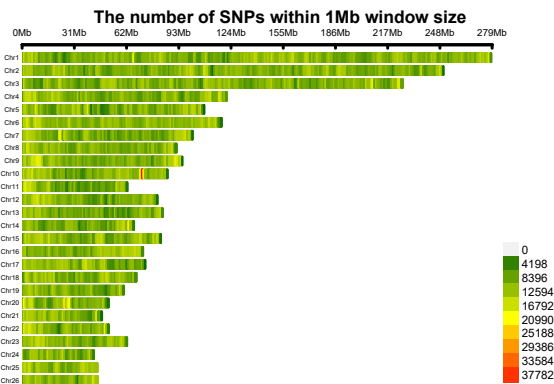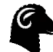

B

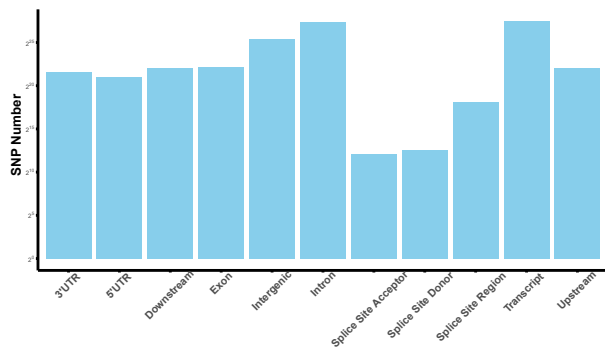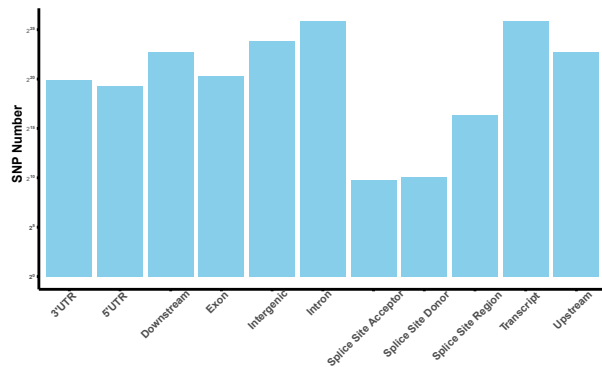

Supplement: Supplementary file 2 — Additional file 2: Fig. S1. Characterization of SNP variants in goats and sheep. A Distribution of SNPs within 1 Mb sliding windows across goat and sheep autosomes. B SNP number distribution across different genomic regions in goats and sheep. [file 40104_2025_1334_MOESM2_ESM.pdf]

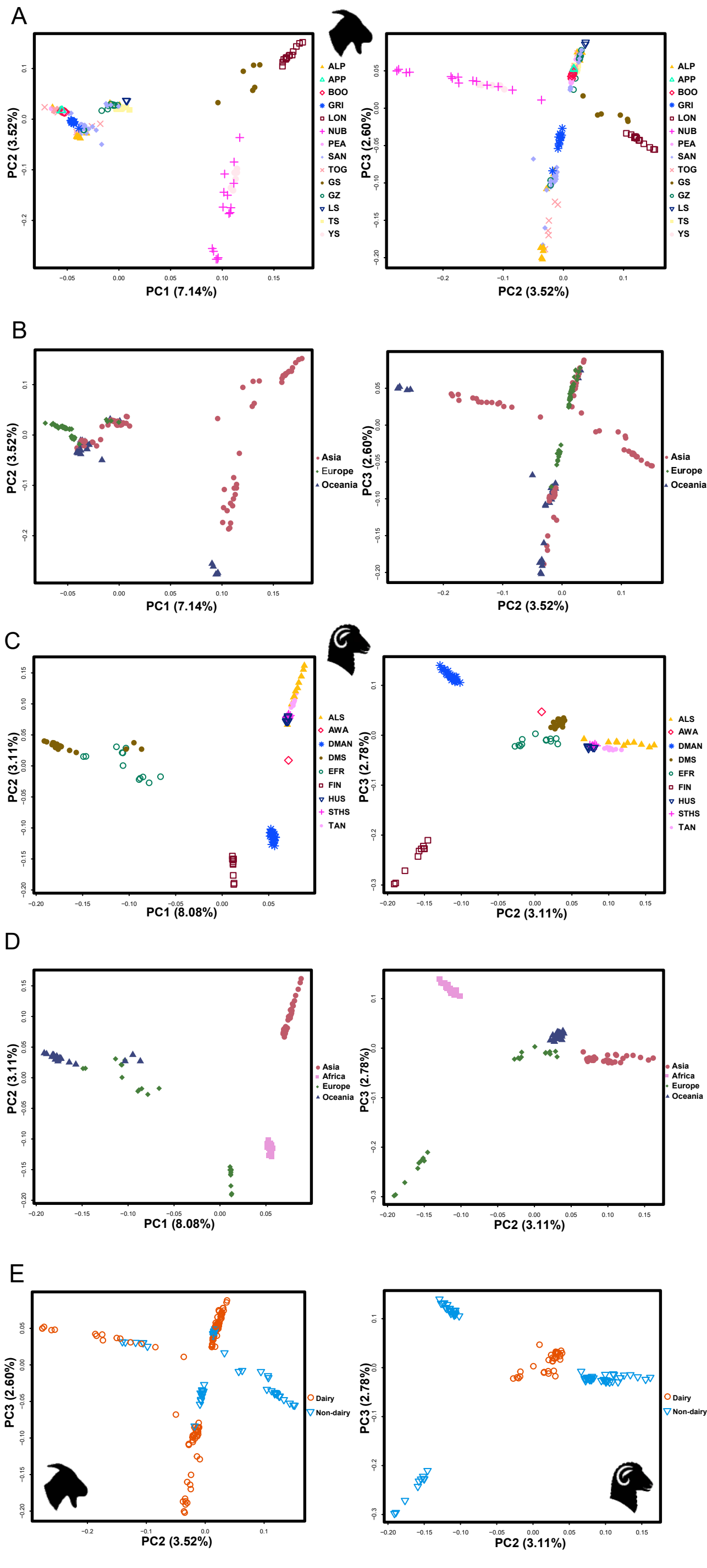

Supplement: Supplementary file 3 — Additional file 3: Fig. S2. Principal component analysis (PCA) of goat and sheep breeds. A PCA of different goat breeds. B PCA of goat populations grouped by geographic origin (Asia, Europe, Oceania). C PCA of different sheep breeds. D PCA of sheep populations grouped by geographic origin (Asia, Africa, Europe, Oceania). E PCA of dairy and non-dairy goat and sheep populations. [file 40104_2025_1334_MOESM3_ESM.pdf]

A

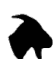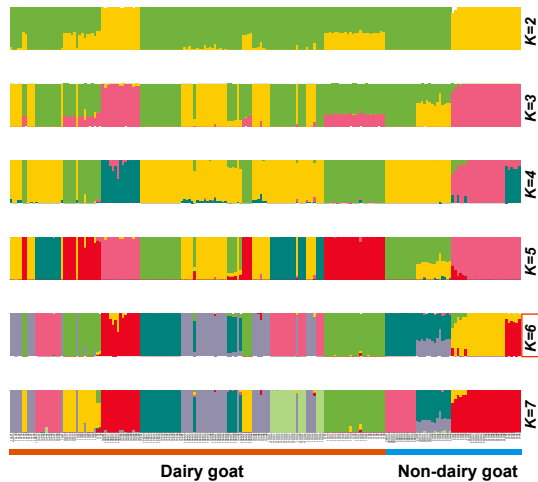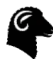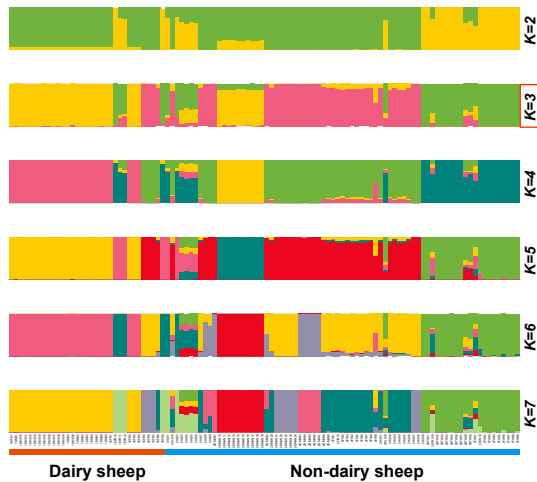

B

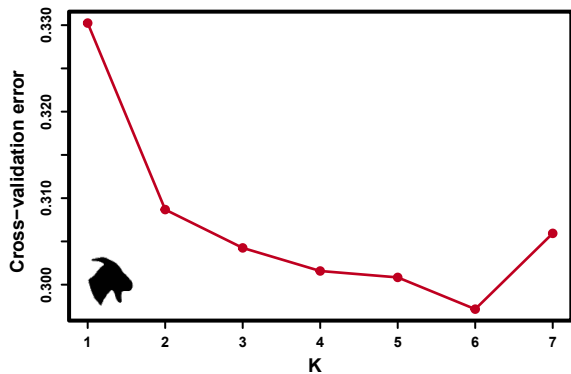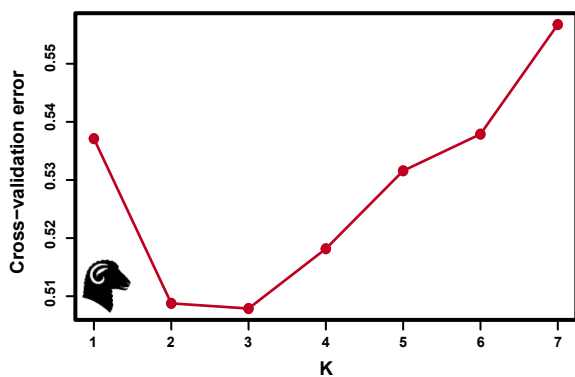

Supplement: Supplementary file 4 — Additional file 4: Fig. S3. Population structure analysis of dairy and non-dairy goats and sheep. A ADMIXTURE analysis of dairy and non-dairy goats and sheep at K = 2–7. B Cross-validation error values for different K clusters in goats and sheep. [file 40104_2025_1334_MOESM4_ESM.pdf]

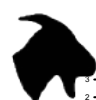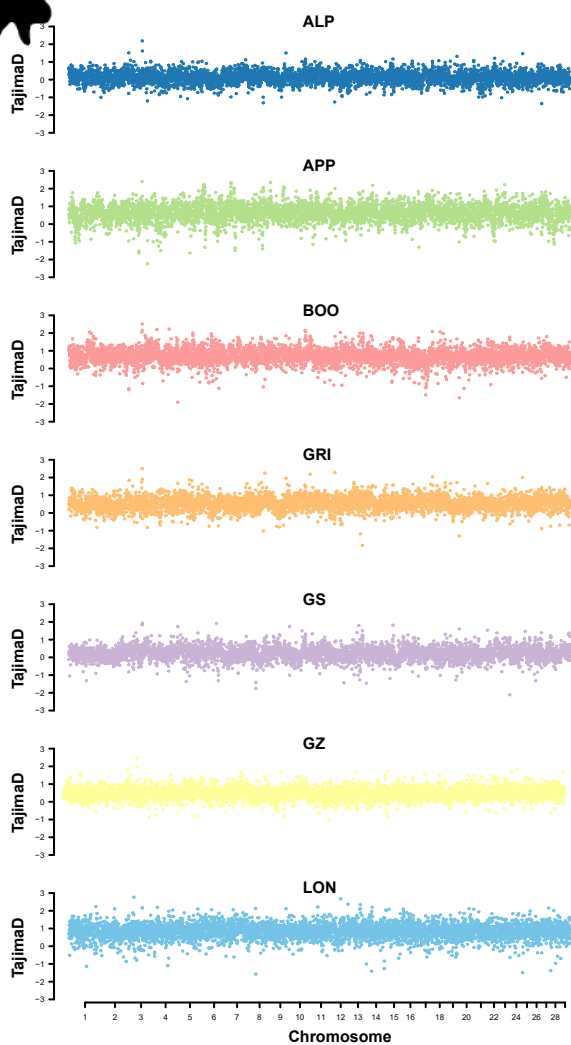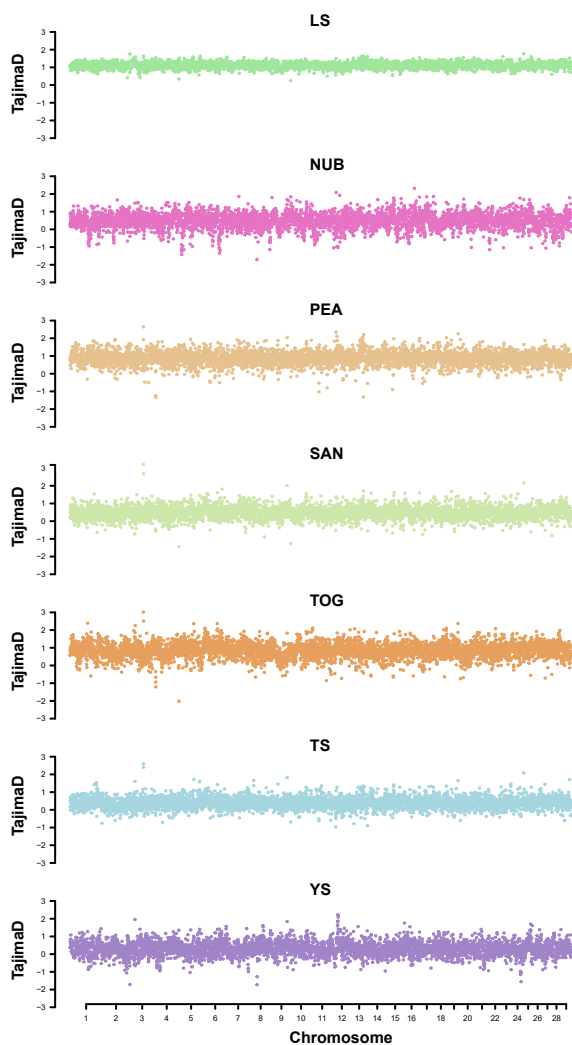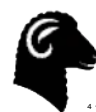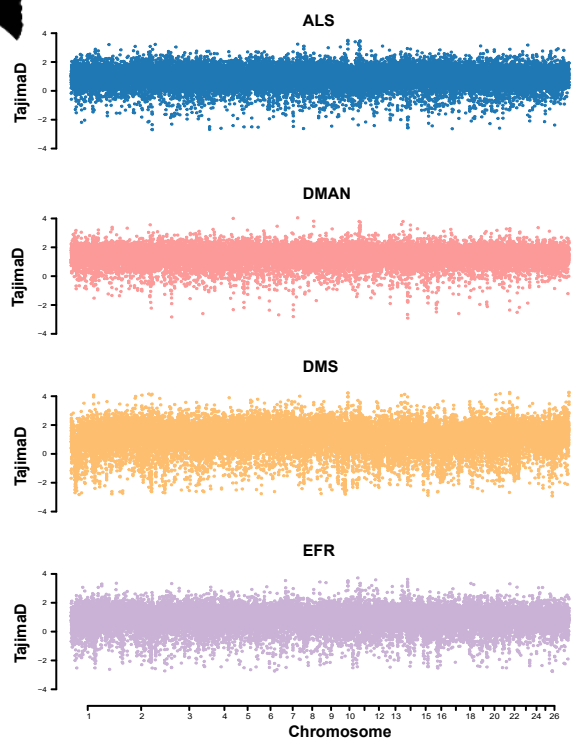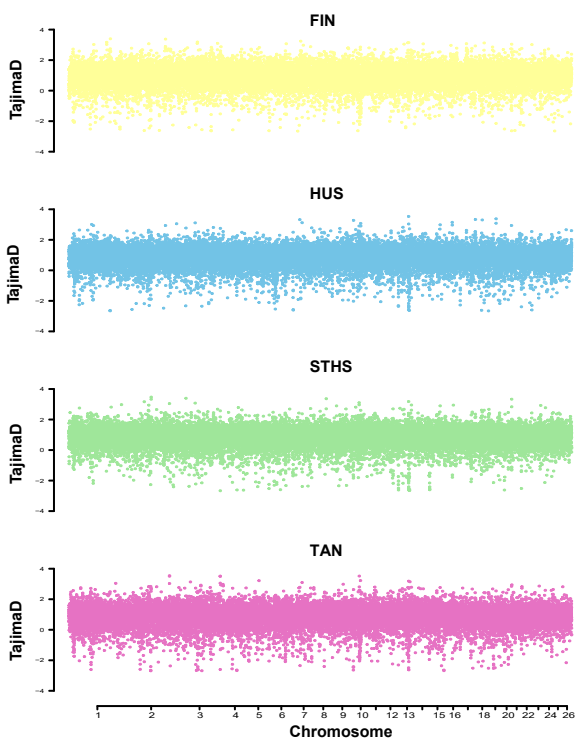

Supplement: Supplementary file 5 — Additional file 5: Fig. S4. Tajima’s D values in different goat and sheep breeds. [file 40104_2025_1334_MOESM5_ESM.pdf]

Terms

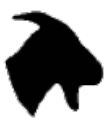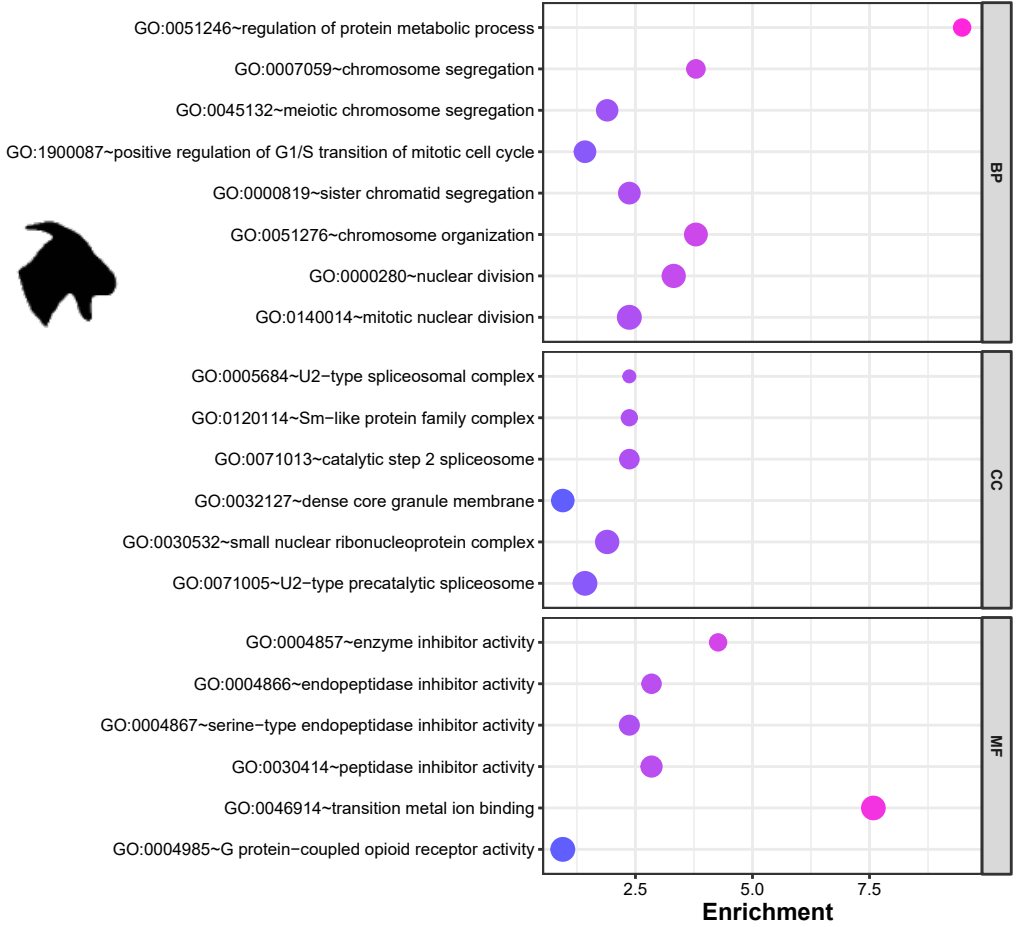

Terms

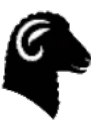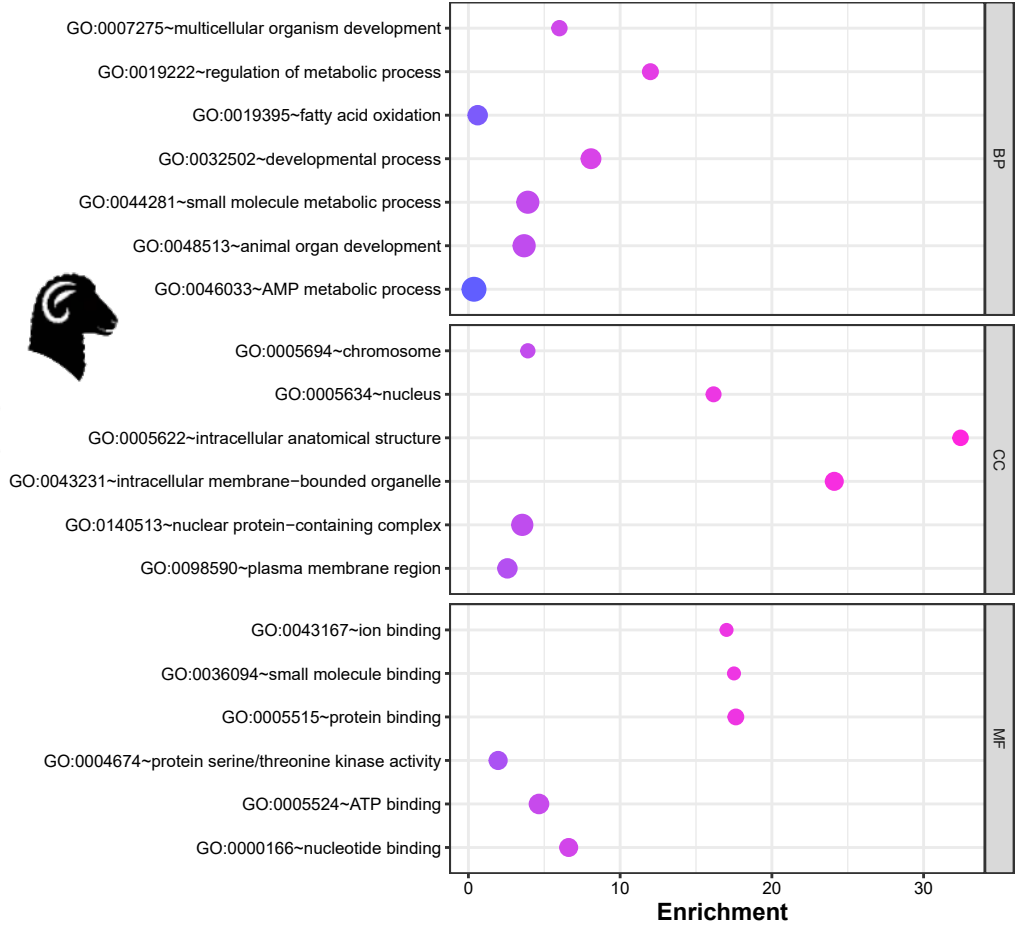

Supplement: Supplementary file 6 — Additional file 6: Fig. S5. Gene Ontology (GO) enrichment analysis of goats and sheep. [file 40104_2025_1334_MOESM6_ESM.pdf]

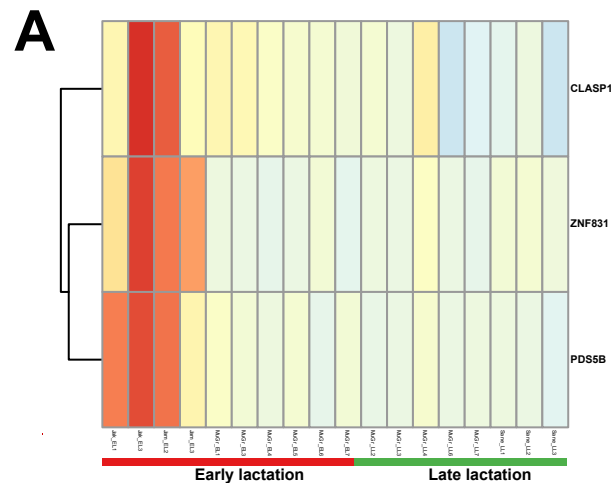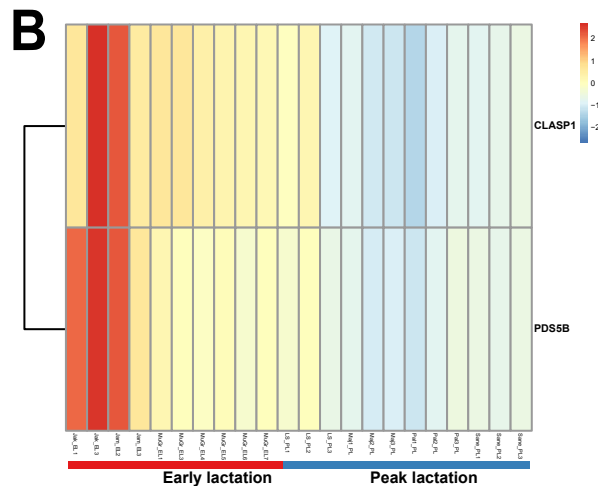

Supplement: Supplementary file 7 — Additional file 7: Fig. S6. Haplotype block structures of candidate genes in dairy and non-dairy breeds. A Haplotype blocks of PDS5B in goats and sheep. B Haplotype blocks of ZNF831 in goats and sheep. C Haplotype blocks of CCDC73 in goats and sheep. [file 40104_2025_1334_MOESM7_ESM.pdf]

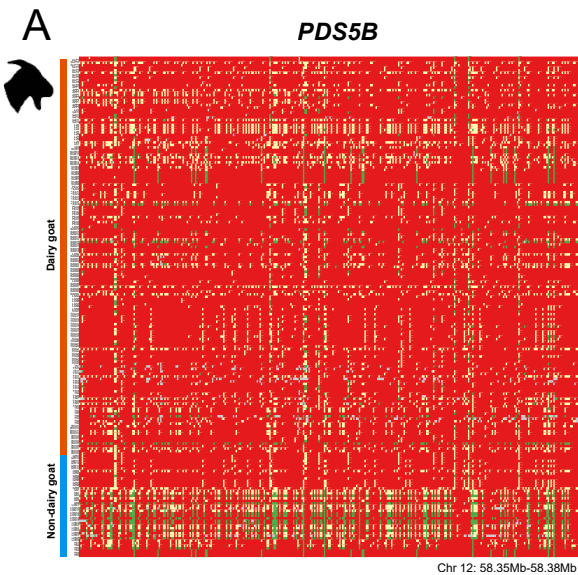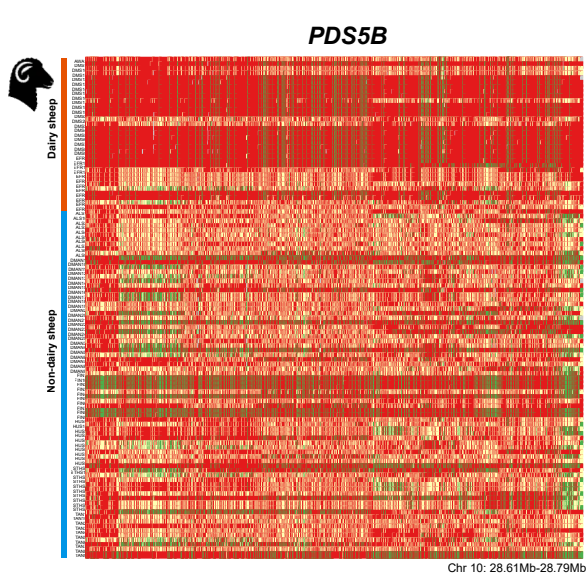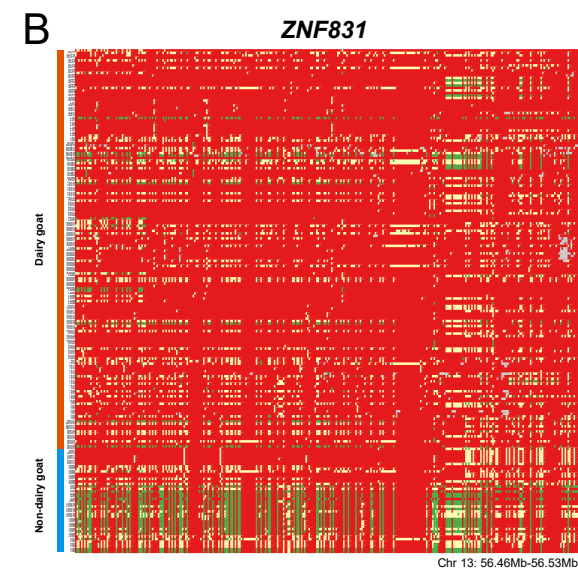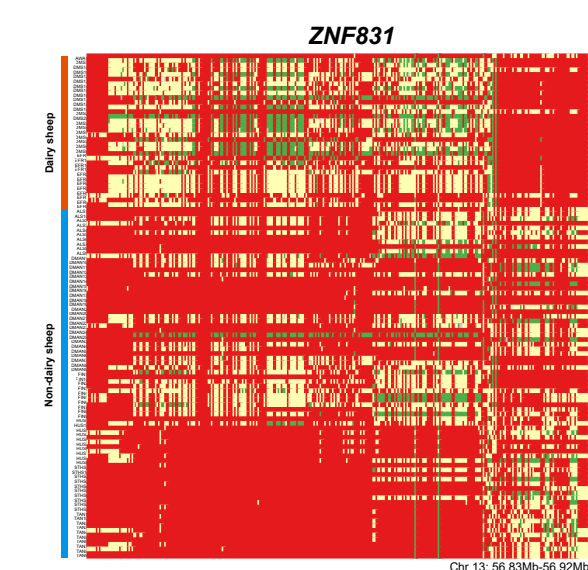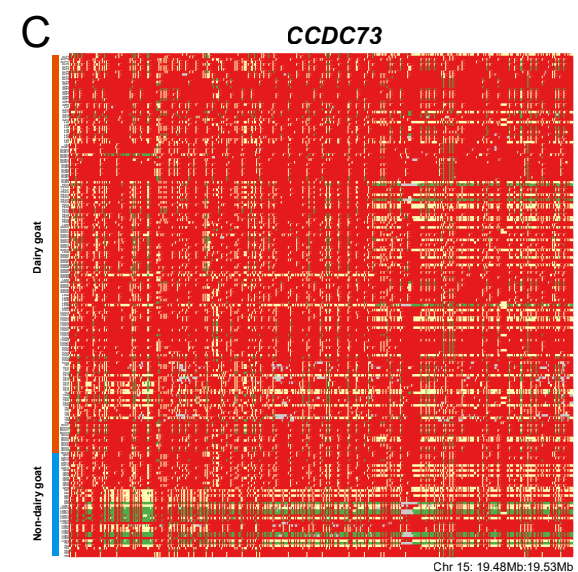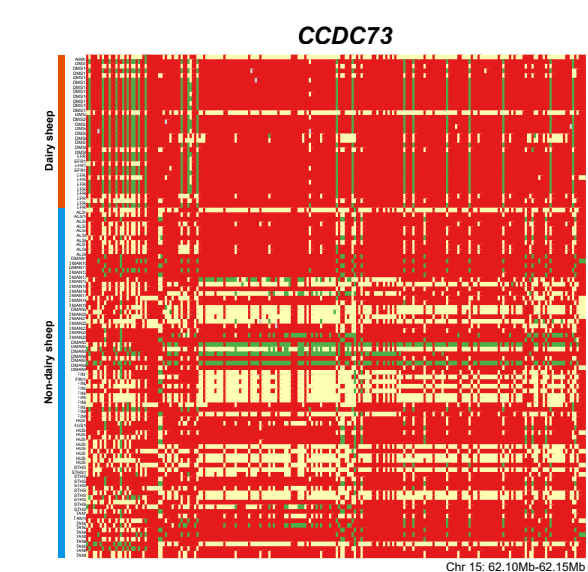

Supplement: Supplementary file 8 — Additional file 8: Fig. S7. Transcriptomic analysis of goat samples during different lactation stages. A Heatmap of candidate gene expressions between early and late lactation in goats. B Heatmap of candidate gene expressions in early vs. peak lactation in goats. C Volcano plot of DEGs between early and late lactation in goats. D Volcano plot of DEGs between early and peak lactation in goats. [file 40104_2025_1334_MOESM8_ESM.pdf]
